# Randomized study of the effects of testosterone administration on severity of asthma in horses

**DOI:** 10.1093/jvimsj/aalag187

**Published:** 2026-09-07

**Authors:** Camille Ruault, Sophie Mainguy-Seers, Laurence Leduc, Tristan Juette, Guillaume St-Jean, Jean-Pierre Lavoie

**Affiliations:** Department of Clinical Sciences, Faculty of Veterinary Medicine, Université de Montréal, Saint-Hyacinthe, Quebec, Canada; Department of Clinical Sciences, Faculty of Veterinary Medicine, Université de Montréal, Saint-Hyacinthe, Quebec, Canada; Department of Clinical Sciences, Faculty of Veterinary Medicine, Université de Montréal, Saint-Hyacinthe, Quebec, Canada; Faculty of Veterinary Medicine, Université de Montréal, Saint-Hyacinthe, Quebec, Canada; Department of Pathology and Microbiology, Faculty of Veterinary Medicine, Université de Montréal, Saint-Hyacinthe, Quebec, Canada; Department of Clinical Sciences, Faculty of Veterinary Medicine, Université de Montréal, Saint-Hyacinthe, Quebec, Canada

**Keywords:** androgen, bronchial remodeling, heaves, horse, lung function

## Abstract

**Background:**

Low testosterone concentrations are associated with poor lung function in human asthmatics, and androgen administration improves airway obstruction in women with asthma.

**Hypothesis/Objectives:**

Testosterone improves clinical scores, lung function, airway neutrophilia, and airway remodeling in horses with severe asthma.

**Animals:**

Ten horses affected by severe asthma from a research herd.

**Methods:**

In a randomized, blinded, crossover study, horses received dexamethasone (0.06 mg/kg PO q24h for 10 days) or a single intramuscular injection of testosterone cypionate (0.35 mg/kg), with a 2-week washout period. Clinical respiratory scores were assessed at days 0, 2, 5, and 10; lung function was evaluated at days 0, 5, and 10; and bronchoalveolar lavage fluid cytology and central airway remodeling at days 0 and 10. Mixed linear models were used for analysis.

**Results:**

The respiratory clinical scores improved with dexamethasone administration (median [95% CI], from 12 (9-13) to 5 (3-6), *P* < .001), and remained unchanged with testosterone administration (from 10 [8-12] to 9 [8-11], *P* = .6). Pulmonary elastance improved with both treatments, but the effect was more pronounced with dexamethasone administration (from 5.1 cm H_2_O/L [2.4-5.8] to 0.5 [0.4-0.8], *P* < .001) than with testosterone administration (from 4.5 cm H_2_O/L [1.6-8.5] to 3.0 [1.0-4.1], *P* = .04). Pleural pressure and pulmonary resistance decreased only with dexamethasone. Neither treatment altered airway neutrophilia or bronchial remodeling.

**Conclusions and clinical importance:**

Testosterone is less effective than dexamethasone and only mildly improves pulmonary elastance in horses with severe asthma.

## Introduction

Severe equine asthma is a chronic pulmonary disease affecting approximately 15% of horses in North America.^1^ It is characterized by a partially reversible bronchial obstruction resulting from bronchospasm, mucus accumulation in the airways, and thickening and inflammation of the bronchial walls.^2^^,^^3^ Although the pathogenesis is not fully understood, asthma in horses shares several features with human asthma,^3^^,^^4^ involving complex interactions between environmental, genetic, and physiological factors that influence disease severity.^3^^,^^5^

In humans, asthma is more prevalent in boys during childhood, whereas this predominance reverses after puberty,^6–9^ suggesting that male sex hormones might have protective effects. Furthermore, high testosterone concentrations are associated with a lower asthma prevalence and severity, especially in men, in whom high androgen concentrations are associated with better lung function.^10^ Nebulized dehydroepiandrosterone (DHEA)-sulfate, a sex hormone precursor, administered over 6 weeks to human patients with moderate to severe asthma leads to improvements in clinical symptoms and reductions in asthma-related limitations on daily activities.^11^ Although the exact mechanisms have yet to be elucidated, the beneficial effects of androgens in asthma might involve the relaxation of bronchial smooth muscle and anti-inflammatory actions.^12^

In horses, little is known regarding the effects of sex hormones on the ontogeny and severity of asthma. Mares are slightly more likely to be diagnosed with severe asthma than stallions,^13^ and the estrous cycle can influence the severity of airway obstruction.^14^ In this proof-of-concept study, we hypothesized that testosterone administration would improve clinical respiratory scores and lung function in horses with severe asthma. Our objectives were to compare the effects of injectable testosterone cypionate with those of oral dexamethasone on clinical scores, lung function, airway neutrophilia, and central bronchial remodeling in severe asthma in horses, and to describe their effect over time on these variables.

## Materials and methods

All experimental procedures were performed in accordance with the Canadian Council for Animal Care guidelines and were approved by the Animal Care Committee of the Faculty of Veterinary Medicine of the Université de Montréal (Protocol #23-Rech-2254, October 10, 2023).

### Animals and housing

Ten horses (6 mares and 4 geldings) from a research herd were studied. They were mixed breeds, aged 19 ± 3 years (mean ± SD), and weighed 546 ± 69 kg.

To standardize management conditions and induce clinical exacerbation, horses were housed individually, fed hay, and given daily access to group turnouts in dry paddocks for 1 month before the beginning of the study. The environmental and dietary conditions remained unchanged during the treatment phases. Endpoints included inappetence, reduced manure production, colic, hyperthermia, respiratory distress, or any other medical conditions necessitating treatment. Respiratory difficulty associated with tachycardia over 60 bpm or reduced feed consumption triggered sporadic inhaled bronchodilator (β2-agonist) administration.

### Study design

Using a randomized controlled crossover design (Figure S1), horses were allocated to receive either a single intramuscular dose of testosterone as the initial treatment (testosterone cypionate; Taro Pharmaceutical Inc., Brampton, Ontario; 0.35 mg/kg) at day 0, or dexamethasone (dexamethasone powder; Dominion Veterinary Laboratories Ltd, Longueuil, Quebec; 0.06 mg/kg PO), mixed with molasses, once a day for 10 days. Group allocation was based on lung resistance (*R*_L_) ranking values, and treatment order was randomized. Reported testosterone doses in equine studies range from 0.15 mg/kg^15^ to 1 mg/kg.^16^ We selected a dose within this range based on anecdotal reports of clinical effectiveness and a 2-week withdrawal period between both treatment phases (providing 28 days after the single injection of testosterone), which was informed by a pharmacokinetic study of an aqueous form of testosterone in which plasma testosterone concentrations decreased below the detection threshold after 28 days.^17^ To maintain evaluators’ blinding, horses receiving dexamethasone orally also received intramuscular sterile saline (2 mL), and horses receiving testosterone cypionate received 5 mL of molasses orally daily.

## Results

All the horses tolerated both drugs well, with no notable adverse effects other than behavioral changes. There was no significant variation in the body weight of the horses during the study (*P* = .5). Occasional rescue bronchodilator therapy with salbutamol (Ventolin, GlaxoSmithKline Inc., Mississauga, Ontario; 2 μg/kg once daily as needed) was required in 3 horses experiencing marked airway obstruction—2 during the pretreatment exposure phase and 1 early in the dexamethasone phase.

### Clinical scores

**Figure 1:**
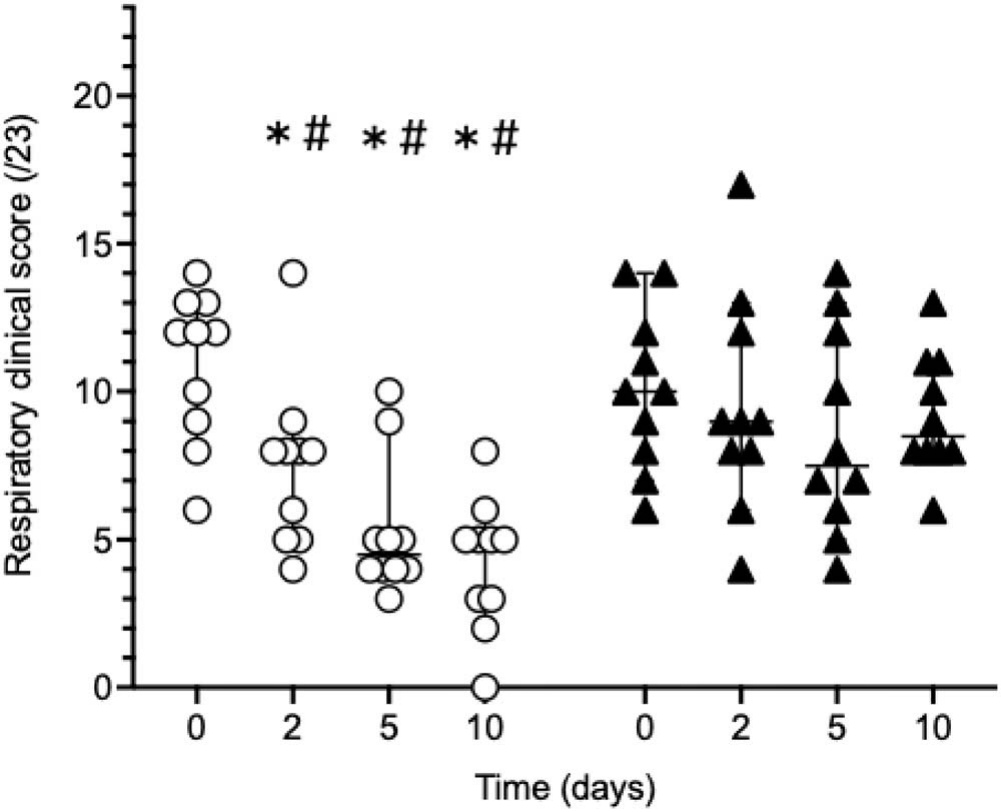
Respiratory clinical score (dot plot with the median with 95% CI) in horses with severe asthma treated with dexamethasone (blank circles) and testosterone (full triangles). ^*^, *P* < .05, difference compared to day 0 in the same treatment group; ^#^, *P* < .05, difference between the treatment groups at the same time point.

All horses showed increased abdominal effort, nostril flaring, and a score above 5 after 1 month of antigen exposure, with no difference in baseline respiratory clinical scores before the testosterone cypionate and dexamethasone treatments. A main effect of time of therapy (*P* < .001) and an interaction between time and treatment types (*P* < .001) were observed using the linear model, with no difference between treatment types (*P* = .6). Multiple comparisons indicated that, in comparison to testosterone cypionate, only dexamethasone improved the clinical score over time (Figure 1; Table S1; dexamethasone [median (95% CI), from 12 (9-13) to 5 (3-6), *P* < .001], testosterone [from 10 (8-12) to 9 (8-11), *P* = .6]). There was a negative correlation between serum testosterone concentrations and the clinical score on days 5 (*r* = −0.8, *P* = .01) and 10 (*r* = −0.7, *P* = .03), indicating that horses with higher testosterone concentrations exhibited fewer clinical signs during testosterone treatment.

### Lung function

All horses had developed airway obstruction at baseline (*P*_L_ values > 15 cm H₂O and *R*_L_ and *E*_L_ values > 1 cm H₂O/L/s and > 1 cm H₂O/L, respectively), and there was no difference between the baseline values before the testosterone cypionate and dexamethasone treatments.

**Figure 2:**
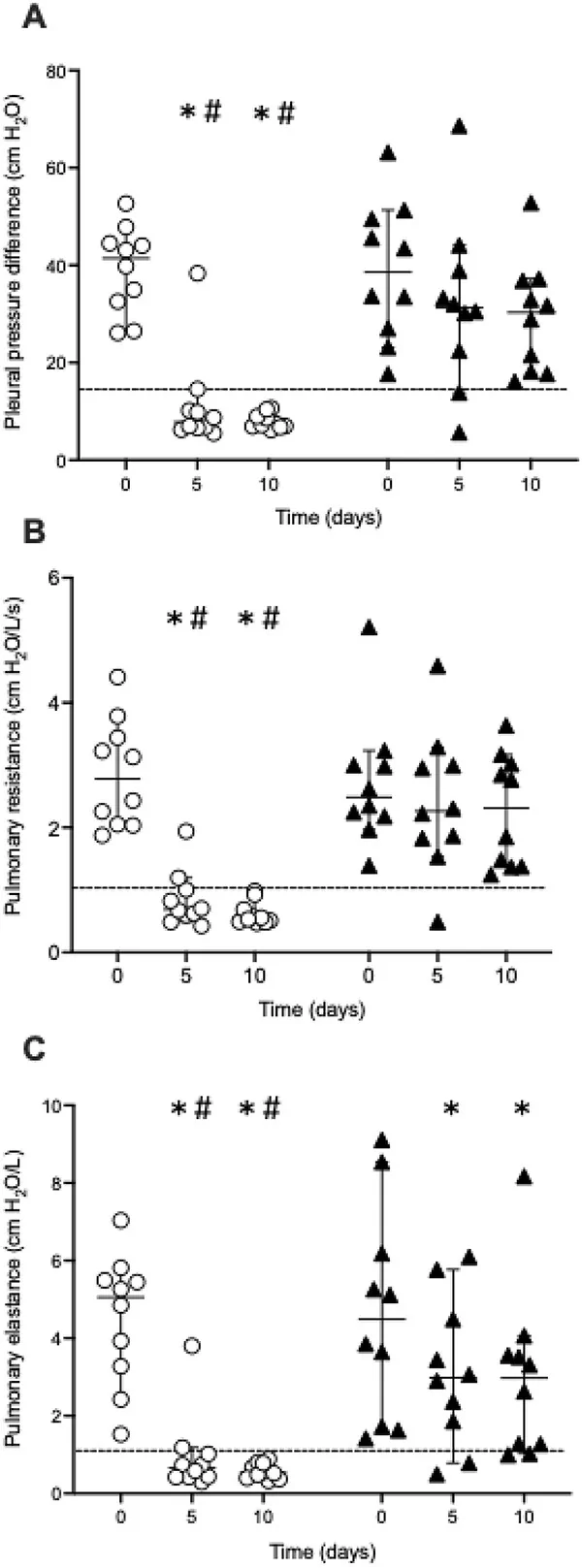
Lung function (dot plot with the median with 95% CI) in horse with severe asthma treated with dexamethasone (blank circles) and testosterone (full triangles). (A) Pleural pressure difference, (B) pulmonary resistance, and (C) pulmonary elastance. ^*^, *P* < .05, difference compared to baseline values (day 0) in the same treatment group; ^#^, *P* < .05, difference between the treatment groups at the same time point. The dotted line corresponds to the value above which horses are considered in exacerbation.

There was a significant main effect of time of therapy (*P* < .001) in *P*_L_, *R*_L_, and *E*_L_ and an interaction between time and treatment types (*P* < .01 for *P*_L_ and *R*_L_ and *P* = .04 for *E*_L_) with better lung function values at days 5 and 10 when horses were treated with dexamethasone compared to testosterone (Figure 2). In the multiple comparisons evaluating the effect of therapy over time, dexamethasone was the only treatment that decreased *P*_L_ and *R*_L_, whereas both treatments improved elastance, with a greater improvement observed with the corticosteroid (Figure 2; Table S1; dexamethasone [from 5.1 cm H_2_O/L (2.4-5.8) to 0.5 (0.4-0.8), *P* < .001], testosterone [from 4.5 cm H_2_O/L (1.6-8.5) to 3.0 (1.0-4.1), *P* = .04]). There were no correlations between lung function variables and testosterone serum concentrations.

### Airway inflammation

There was no significant effect of time, treatment, and order of treatment on the percentage of neutrophils, macrophages, lymphocytes, mast cells, and eosinophils in the bronchoalveolar lavage fluid (BALF) (Table 1). The total number of cells in the BALF was higher on day 10 with dexamethasone (*P* = .05; Table 1) than with testosterone, but there was no variation over time (*P* = .9).

**Table 1:** Bronchoalveolar lavage fluid cytology.

	Testosterone		Dexamethasone	
Time	Day 0	Day 10	Day 0	Day 10
**Total cells (cells/μL)**	324.6 ± 114.9	230.8 ± 41.5^a^	300.8 ± 63.5	321 ± 36.6^a^
**Neutrophils (%)**	19.5 (±6)	14.9 (±3.4)	23.7 (±8.5)	23.2 (±8.2)
**Mast cells (%)**	0.6 (±0.2)	1.2 (±0.4)	0.5 (± 0.1)	0.75 (±0.2)
**Lymphocytes (%)**	42.1 (±3.9)	50.3 (±3.2)	40.2 (±5.9)	42.5 (±5.3)
**Macrophages (%)**	25.6 (±3)	30.2 (±2.1)	25.3 (±4.2)	24.7 (±3.8)
**Eosinophils (%)**	0.02 (±0.02)	0.17 (±0.1)	0.1 (±0.08)	0.05 (±0.03)

^a^ *P* < .05, difference between groups at the same time point. Data are presented as mean ± SD.

### Endobronchial biopsies

**Figure 3:**
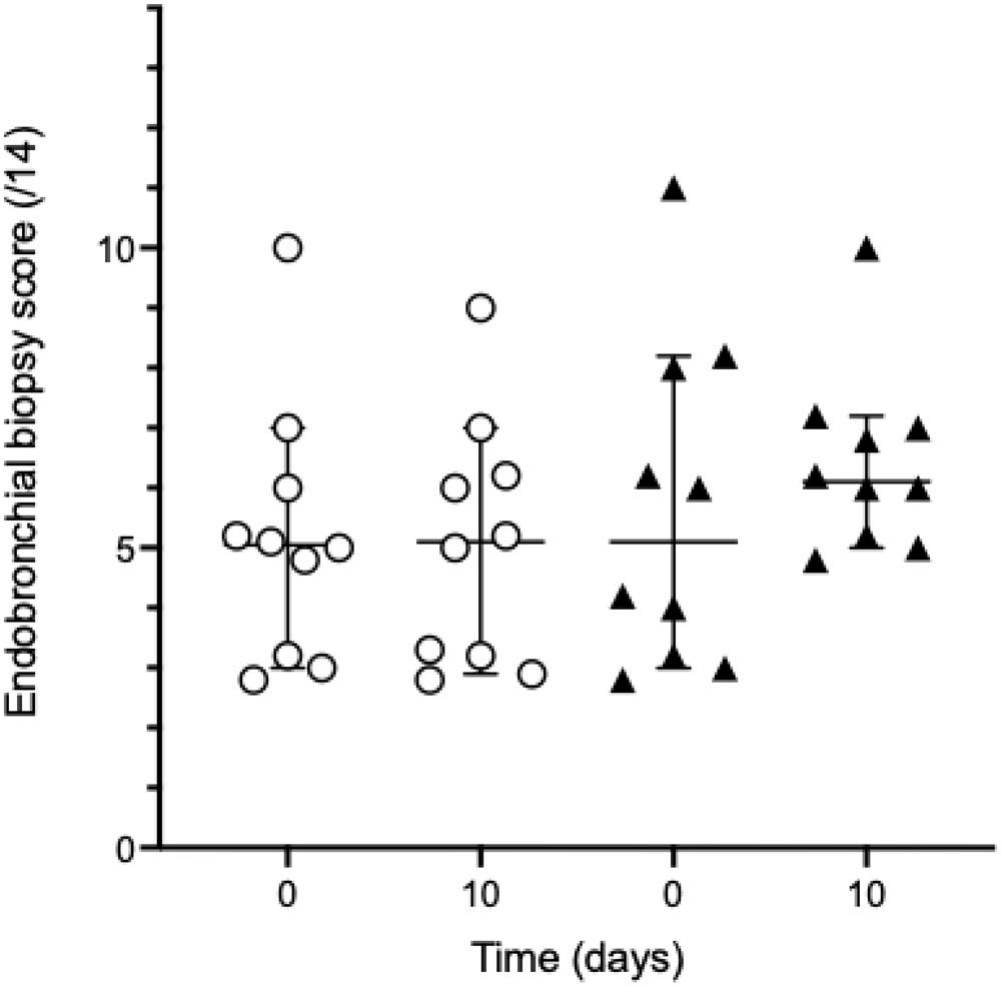
Endobronchial biopsy score (dot plot with the median with 95% CI) in 10 horses with severe asthma treated with dexamethasone (blank circles) and testosterone (full triangles).

There was no difference in airway remodeling scores between treatments at baseline. The scores remained unchanged over time (*P* = .5) and showed no difference between treatments (*P* = .2; Figure 3).

### Serum testosterone concentrations

**Figure 4:**
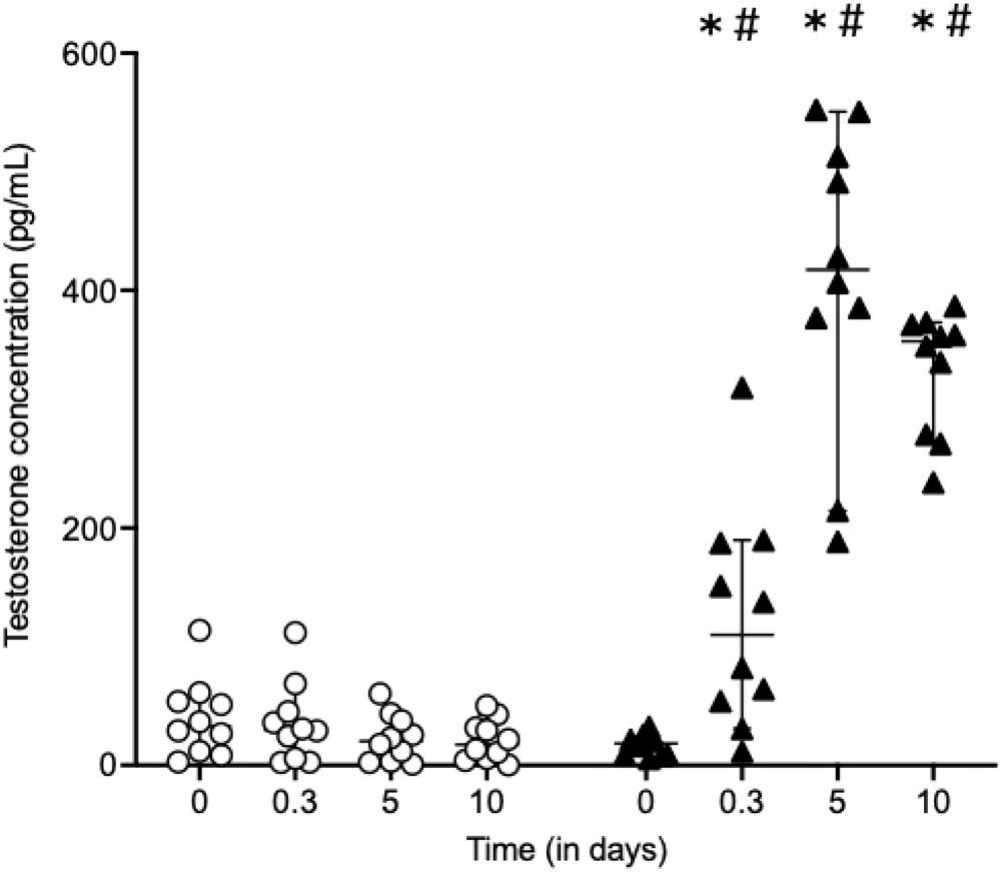
Serum testosterone (dot plot with the median with 95% CI) in 10 horses with severe asthma treated with dexamethasone (blank circles) and testosterone (full triangles). ^*^, *P* < .05, difference compared to baseline (day 0) in the same treatment group; ^#^, *P* < .05, difference between the treatment groups at the same time point.

There was a significant treatment order effect (*P* = .001) on serum testosterone concentrations, as well as a significant interaction between time and treatment (*P* < .001). The testosterone concentration significantly increased from 16.9 ± 8.9 pg/mL (mean ± SD) at baseline to 333.5 ± 51.4 pg/mL at day 10, reaching a peak of 410.8 ± 127.6 pg/mL at day 5 when horses were treated with testosterone cypionate, whereas it remained unchanged during the dexamethasone treatment (Figure 4; Table S1).

Baseline testosterone concentrations were higher in horses receiving dexamethasone in the second treatment phase (56.84 ± 33.4 pg/mL) compared with those treated in the first phase (22.2 ± 23.5 pg/mL, *P* = .006), suggesting incomplete clearance of the administered testosterone.

### Behavior analysis

The non-blinded observer noted obvious aggressive and sexual behaviors (penile erection, flehmen) in all horses after testosterone cypionate administration when they were out in the paddock. These behavioral changes persisted in all horses even after serum concentrations normalized, and one gelding was isolated from the rest of the herd during the study with its turnout time reduced for safety reasons due to aggressiveness toward other horses. These changes were also noticeable to the blind observer in some horses while they were in the stall (aggressivity, penile erection).

**Figure 5:**
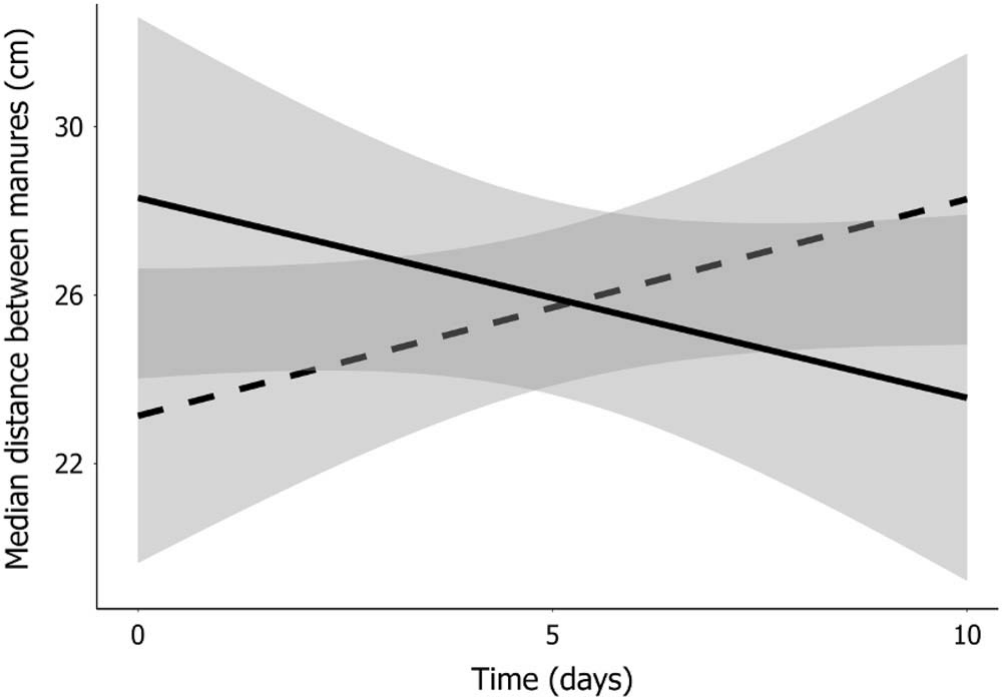
Distance between manure piles (median ± 95% CI) over time in 10 horses with severe asthma treated with dexamethasone (hatched line) or testosterone (solid line).

There was a significant effect of time (*P* = .03) and an interaction between time and treatment (*P* = .04) on the dispersion of manure in the box stalls as it increased significantly over time during dexamethasone treatment (slope for dexamethasone of 0.58 [95% CI, 0.05 to 1.11], slope for testosterone cypionate of −0.22 [95% CI, −0.75 to 0.32]; Figure 5).

For all the studied outcomes, the nonsignificant variables in the mixed generalized linear models are shown in Table 2.

**Table 2:** Nonsignificant effects of explicative variables within the mixed generalized linear models.

Dependent variable	Explicative variable	*P*-value
**Clinical scores**	Treatment	.38
	Order	.93
**Pleural pressure**	Treatment	.76
	Order	.37
**Pulmonary resistance**	Treatment	.63
	Order	.89
**Pulmonary elastance**	Treatment	.65
	Order	.38
**% Neutrophils in bronchoalveolar lavage**	Time	.39
	Treatment	.34
	Order	.33
	Time × Treatment	.95
**Biopsy score**	Time	.50
	Treatment	.16
	Order	.95
	Time × Treatment	.30
**Testosterone serum concentration**	Time	.10
	Treatment	.09
**Distance between manures**	Treatment	.05
	Order	.42

## Discussion

This blinded, randomized, crossover study demonstrated that testosterone administration in horses with severe asthma partially improved pulmonary elastance during clinical exacerbations, consistent with the association between elevated testosterone concentrations and enhanced lung function in humans.^10^ Despite the absence of a significant improvement in the respiratory score over time, the negative correlations between testosterone concentrations and the score at days 5 and 10 suggest a possible beneficial effect on clinical signs. Testosterone cypionate did not reduce airway neutrophilia or bronchial remodeling. Behavioral changes were observed in both geldings and mares treated with testosterone cypionate, and these changes persisted even after serum testosterone concentrations had normalized.

The mechanisms that could have contributed to the improvement in elastance include the non-genomic actions of androgen, mainly the relaxation of airway smooth muscle (ASM),^12^ as described in guinea pigs^18–20^ and rabbits.^21^ If androgens exert beneficial effects via ASM bronchodilation, this would provide a plausible explanation for the selective improvement in *E*_L,_ which reflects peripheral airway obstruction,^22^ the site of the most pronounced ASM enlargement in severe asthma in horses.^23^ Measuring lung function within the first hours of treatment would have helped confirm the contribution of non-genomic bronchodilation, as these effects occur more rapidly than genomic mechanisms. The improvement in *E*_L_ could also be explained by a decrease in peripheral ASM mass. The inhibition of bronchial smooth muscle migration observed in vitro with DHEA-sulfate,^24^ could have contributed to such a reduction. However, this aspect could not be specifically assessed in the current study as endobronchial biopsies sample central airways and do not allow the quantification of ASM mass.^25^ Androgens could also have improved the *E*_L_ by reducing fibroblast proliferation and collagen deposition in the airway extracellular matrix. Collagen withstands tensile forces, and its quantity is increased in the peripheral asthmatic airways, which contributes to airway stiffening.^26–28^ In interstitial pulmonary fibrosis, low DHEA plasma concentrations are associated with poorer lung function.^29^ Androgens exert anti-fibrotic effects by decreasing the expression of growth factors associated with collagen synthesis and by inhibiting both fibroblast proliferation and mesenchymal transition.^30^^,^^31^ While testosterone might affect ASM and collagen remodeling, the rapid improvement in lung elastance within 5 days suggests a predominantly functional effect, similar to that of a bronchodilator, rather than structural changes.

Dexamethasone normalized lung function and improved the clinical signs, demonstrating the reversibility of bronchial obstruction in these horses. Thus, the limited response to testosterone cypionate cannot be explained by irreversible airway obstruction in these animals. This study reports the effects of a short dexamethasone treatment on central airway remodeling. The lack of change in the endobronchial biopsy score after 10 days of dexamethasone treatment suggests that a longer treatment duration, similar to that reported for inhaled corticosteroids,^32–34^ might be needed to improve bronchial remodeling.

Male sex hormones have multiple anti-inflammatory effects on the respiratory system,^22^^,^^35–45^ including reducing group 2 innate lymphoid cells, inflammatory cytokines (including some incriminated in horses with asthma such as IL-5, IL-13, and Th17),^5^ and inflammatory cell populations including neutrophils and mast cells.^12^^,^^42^^,^^43^ However, no reduction of inflammation was observed in our study. The use of BALF leukocytes as a coarse marker of inflammation could have failed to capture the full spectrum of androgen-mediated immunomodulatory effects, as is the case with dexamethasone, which does not normalize airway neutrophilia but reduces neutrophil activation^46^ and several inflammatory cytokines in pulmonary tissues.^47^

Reproductive behaviors and aggressiveness were observed in these normally calm geldings and mares after administration of testosterone cypionate, and these behaviors persisted even after serum testosterone concentrations returned to normal. This suggests that even small changes in testosterone concentrations might influence behavior. We also monitored manure pile dispersion within the stall, which has been described as a stallion-like behavior pattern,^48^ but it remained unchanged with testosterone treatment. The increased manure dispersion observed in the box stalls of dexamethasone-treated horses is a novel finding that might indicate increased motor activity, potentially reflecting improved respiratory comfort.

The limitations of this clinical trial include the small number of horses studied, which was somewhat mitigated by the crossover design, the homogeneity of environmental conditions in the research setting, and the extensive characterization of these horses. Testosterone cypionate is the only testosterone formulation currently available in Canada. Our results show that it is well tolerated and leads to serum testosterone concentrations similar to those usually seen in stallions.^49^ Still, it remains unknown whether serum testosterone concentration accurately reflects pulmonary concentrations. The use of DHEA-sulfate would have been preferred because its effects seem to be greater than those of testosterone in humans,^50^ it has good oral bioavailability in racehorses,^51^ and its nebulization resulted in improved clinical signs in human asthmatics.^11^ However, DHEA is banned in Canada because of its anabolic and doping properties. Another limitation was the insufficient washout period after testosterone cypionate administration, as suggested by persistent behavioral changes and differences in baseline serum testosterone concentrations before dexamethasone treatment. This raises the possibility of residual testosterone effects influencing outcomes in the second treatment period, including behavioral assessments. However, no significant baseline differences were observed between the 2 study periods across measured variables. Furthermore, if present, any residual beneficial effect of testosterone would have been expected to enhance the apparent respiratory response to dexamethasone during the second phase, which was not observed. Finally, another limitation is that the power calculation was performed on *R*_L_ as an indicator of central bronchodilation, whereas the only lung function variable that improved with testosterone administration was *E*_L_. Although both variables typically improve with standard asthma therapy, the magnitude of the changes could differ because they reflect different components of lung mechanics.

Our findings suggest that androgens might contribute to the complex pathogenesis of severe asthma in horses and raise the question of whether castration could influence the long-term progression of this chronic disease in horses. Although a modest improvement in peripheral airway obstruction was observed with testosterone cypionate, it should not be used as a therapeutic option for severe asthma in horses due to its limited efficacy, behavioral effects, and doping potential.

## Supplementary Material

SUPPLEMENTARY_FIGURE-TABLE-ready_for_publication_aalag187

## Off-label antimicrobial declaration

The authors declare no off-label use of antimicrobials.

## Institutional animal care and use committee or other approval declaration

All experimental procedures were performed in accordance with the Canadian Council on Animal Care guidelines and were approved by the Animal Care Committee of the Faculty of Veterinary Medicine of the Université de Montréal (Protocol # 23-Rech-2254, October 10, 2023).

## Appendix 1

### Animals and housing

The diagnosis of severe asthma was confirmed upon admission in the research herd based on the history of recurrent episodes of respiratory distress when exposed to hay, lung function (maximal pleural pressure difference [*P*_L_] > 15 cm of H_2_O), and pulmonary inflammation on bronchoalveolar lavage fluid (BALF) cytology (>20% neutrophils).^3^ A physical examination, CBC, and serum chemistry were performed before the study to exclude comorbidities.

### Clinical evaluation

A 23-point weighted respiratory clinical score evaluating respiratory rate, nasal discharge, abdominal effort, nostril-flaring, tracheal rales, pulmonary crackles and wheezing, and cough was performed on days 0, 2, 5, and 10 during each treatment period by a single blinded observer.^52^ Horses were weighed at the beginning and end of each treatment phase.

### Lung function

Lung function was measured at baseline and on days 5 and 10 of each treatment phase. Standard lung function measurements were performed in standing unsedated horses as previously described.^53^ In brief, flow rates were determined by a heated pneumotachograph connected to a differential pressure transducer, placed on a mask covering the horse’s nostrils. The esophageal pressure was obtained with an inflated balloon at the tip of a polyethylene catheter positioned in the distal esophagus of the horse, and was used as an index of the *P*_L_. Electronic integration of the flow and pressure signals (Flexiware 7.6, SCIREQ, Montréal, Quebec, Canada) allowed the calculation of lung resistance (*R*_L_) and elastance (*E*_L_) using the multiple regression equation (*P*_L_ = *E*_L_*V* + *R*_L_*V̇* + *K*, where *V* is volume, *V̇* is airflow, and *K* is the transpulmonary end-expiratory pressure). A minimum of 10 valid breaths per 2-min recording were used for analysis.

### BALF cytology

Airway inflammation was evaluated on days 0 and 10 of each treatment phase. Horses were sedated intravenously with detomidine (Dormosedan; Zoetis, Espoo, Finland; 10-20 μg/kg, IV) and butorphanol (Torbugesic; Zoetis, Girona, Spain; 10-20 μg/kg, IV). Bronchial carinas were anesthetized by the topical application of lidocaine hydrochloride 0.5% (Lidocaine Hydrochloride injection USP, Hikma, Mississauga, Ontario) during bronchoscopy (1.6-m video endoscope, 12.8 mm external diameter, Evis Exera II CV-180; Olympus Canada, Richmond Hill, Ontario, Canada). Two 250 mL boluses of warm sterile isotonic saline were sequentially instilled into a main bronchus and then aspirated using a suction pump. The samples were maintained on ice until processed within 120 min. Cytocentrifuged preparations of BALF were stained with a modified Wright-Giemsa stain (Diff-Quick, Fisher Scientific, Waltham, MA, USA). Assessment of cell concentration and viability were performed by an automated fluorescence cell counter (ADAM automated Cell Counter, Montreal-Biotech Inc., Montreal, Quebec, Canada). Differential leukocyte counts were performed blindly from 400 cells.

### Endobronchial biopsies

Endobronchial biopsies were collected as described,^25^ after the bronchoalveolar lavage procedure in the contralateral lung and after lidocaine hydrochloride instillation (0.5%). Smooth oval forceps (Standard Fenestrated and Smooth, 2.3 m, Olympus Medical Systems, Tokyo, Japan) were used to harvest endobronchial biopsies at the carinas of the main bronchi. Four biopsies were fixed in 10% neutral-buffered formalin for 24-48 h and subsequently embedded in paraffin. Histological sections of 5-μm thickness were stained with hematoxylin–eosin–saffron. Coded slides were assessed blindly under bright-field microscopy by a board-certified pathologist using a semiquantitative score of remodeling lesions.^54^ One or 2 biopsies with the best-maintained architecture were selected for histological evaluation.^25^

### Serum testosterone concentrations

Serum testosterone concentrations were measured at baseline, 7 h after administration, and on days 5 and 10 of each treatment period. After 40 min of sedimentation, the blood was centrifuged at 1509 × *g* (3000 rpm) for 10 min and stored at −80°C until assayed by radioimmunoassay at the Clinical Endocrinology Laboratory of the University of California.^49^

### Behavior analysis

Two different observers recorded the horses’ behaviors during the treatment phases: a blinded observer early in the morning when the horses were in the box stalls and a non-blinded observer while the horses were in the paddock in group turnout. The recorded behaviors included aggression toward other horses or humans, estrus-like signs in females, erection in geldings, flehmen, and vocalizations. Every morning, the animal keeper charted the spatial distribution of manure in each box stall using a grid map, with each manure location marked with a cross.

### Statistical analysis

Using G^*^Power (v.3.1.9.7), a 1-tailed power analysis determined that a sample size of 7 horses total, contributing observations under both treatments, was necessary to detect a 30% improvement in *R*_L_ with testosterone treatment, with an effect size *d_z_* of 1.16, an α of 0.05, a power of 80% and assuming a within-subject correlation of 0.9 (data based on lung function results from a subset of horses included in another study).^55^ Our working hypothesis based on previous studies in humans on testosterone and its derivatives^11^^,^^24^^,^^41^^,^^56–59^ was that testosterone would improve lung function in horses with severe asthma. A 30% improvement was chosen as the target effect size because there is a natural day-to-day variability in *R*_L_ in horses with severe asthma.^60^

Statistical analyses were performed with R version 4.3.1 (R Core Team 2023, Vienna, Austria). For continuous dependent variables, linear mixed models were constructed to evaluate the effects of treatment type (dexamethasone and testosterone cypionate), time of therapy, the interaction between these 2 variables, and the order of treatments. The normality of the residuals from linear mixed models was verified using Shapiro–Wilk tests. When the condition was not met, the dependent variables underwent a log10 transformation. The transformed models then correctly met the assumption of normality of the residuals. For ordinal dependent variables, cumulative link mixed models were constructed to test the effects of treatment type, time of therapy, the interaction between the 2, and the order of therapy. In both cases, the identity of the subjects was integrated into the models as a random factor to account for the nonindependence of repeated measures, thus allowing us to obtain reliable estimates of the variables estimated by the models. For binomial-dependent variables, generalized linear mixed models were used, specifying that the relationship was logistic (link = Logit). For all 3 model types, the outputs were obtained using likelihood ratio tests and the treatment order was included as an explanatory variable. Finally, post-hoc pairwise comparisons were conducted for factors with more than 2 levels in order to identify which specific levels differed, and *P*-values from these comparisons were adjusted using the Benjamini–Hochberg procedure to control the false discovery rate. Associations between physiological variables (lung function and clinical score) and testosterone concentrations during the treatment were assessed with either Pearson or Spearman correlations, according to the distribution of the data. Results were considered statistically significant at *P* < .05. Descriptive data are reported as mean ± SD when normally distributed, and as median with 95% CI when not. Figures were created using Prism software (GraphPad Prism 10.1.0; San Diego, CA, USA).

## Abbreviations

ASM: airway smooth muscle
BALF: bronchoalveolar lavage fluid
D: day
DHEA: dehydroepiandrosterone
*E*_L_: lung elastance
MC: multiple comparisons
*P*_L_: pleural pressure
*R*_L_: lung resistance
