# Supplementary material for: Randomized study of the effects of testosterone administration on severity of asthma in horses
Source: J Vet Intern Med. 2026 Sep 7;40(5):aalag187. doi: 10.1093/jvimsj/aalag187 (PMC13546766; doi:10.1093/jvimsj/aalag187)
Supplement: Supplementary file 1 [file supplementary_figure-table-ready_for_publication_aalag187.docx]

SUPPLEMENTARY

Supplementary figure 1. Study design

Supplementary table 1. Multiple comparison results for lung function parameters, clinical scores, and serum testosterone concentrations over time (D, day) for the two treatment groups (dexamethasone or testosterone).

| **Pleural pressure** | | **Elastance** | | **Resistance** | | **Clinical scores** | | | | | **Testosterone concentrations** | | | | |
| --- | --- | --- | --- | --- | --- | --- | --- | --- | --- | --- | --- | --- | --- | --- | --- |
| **Dexamethasone** | | **Dexamethasone** | | **Dexamethasone** | | **Dexamethasone** | | **Testosterone** | | | **Dexamethasone** | | **Testosterone** | | |
| Comparison | p-value | Comparison | p-value | Comparison | p-value | Comparison | p-value | Comparison | p-value | Comparison | | p-value | Comparison | p-value |  |
| D0 > D5 | **<0.001** | D0 > D5 | **<0.001** | D0 > D5 | **<0.001** | D0 > D2 | **0.001** | D0 = D2 | 0.619 | D0 = D0.3 | | 0.555 | **D0 < D0.3** | **< 0.001** |  |
| D0 > D10 | **<0.001** | D0 > D10 | **<0.001** | D0 > D10 | **<0.001** | D0 > D5 | **<0.001** | D0 = D5 | 0.619 | D0 = D5 | | 0.146 | **D0 < D5** | **< 0.001** |  |
| D5 = D10 | 0.316 | D5 = D10 | 0.560 | D5 = D10 | 0.459 | D0 > D10 | **<0.001** | D0 = D10 | 0.619 | D0 = D10 | | 0.146 | **D0 < D10** | **< 0.001** |  |
| **Testosterone** | | **Testosterone** | | **Testosterone** | | D2 > D5 | **0.023** | D2 = D5 | 0.619 | D0.3 = D5 | | 0.313 | **D0.3 < D5** | **< 0.001** |  |
| Comparison | p-value | Comparison | p-value | Comparison | p-value | D2 > D10 | **0.001** | D2 = D10 | 0.745 | D0.3 = D10 | | 0.313 | **D0.3 < D10** | **< 0.001** |  |
| D0 = D5 | 0.133 | D0 > D5 | **0.037** | D0 = D5 | 0.463 | D5 = D10 | 0.235 | D5 = D10 | 0.619 | D5 = D10 | | 0.928 | D5 = D10 | 0.633 |  |
| D0 = D10 | 0.133 | D0 > D10 | **0.037** | D0 = D10 | 0.449 |  |  |  |  |  | | |  |  |  |
| D5 = D10 | 0.948 | D5 = D10 | 0.826 | D5 = D10 | 0.666 |  |  |  |  |  | |  |  |  |  |

p-value less than 0.05 was considered significant and is shown in bold in the table.
